# Demographic, biochemical, clinical, and cognitive symptom differences between smokers and non-smokers in Chinese older male patients with chronic schizophrenia

**DOI:** 10.1007/s00406-024-01762-7

**Published:** 2024-03-10

**Authors:** Wei Li, Ling Yue, Shifu Xiao

**Affiliations:** 1 Department of Geriatric Psychiatry, Shanghai Mental Health Center, Shanghai Jiao Tong University School of Medicine, Shanghai, 200030 China; 2 Alzheimer’s Disease and Related Disorders Center, Shanghai Jiao Tong University, Shanghai, China

**Keywords:** Schizophrenia, Elderly male, Smoking, Albumin, Negative symptoms

## Abstract

**Background:**

Several studies have suggested that smoking may impair cognitive function and worsen psychiatric symptoms in people with schizophrenia, but the results have not been consistent. There have been few studies to date that have examined the effects of smoking in older men with chronic schizophrenia.

**Methods:**

The participants in our study consisted of 167 order Chinese males with chronic schizophrenia and 359 normal control subjects. We split them into smoking and non-smoking groups based on whether or not they smoked. Second, we compared their differences in terms of general demographic characteristics (such as age, education, body mass index, age of illness onset, and course of disease), disease information (such as hypertension, diabetes, and hyperlipidemia), lifestyle factors (such as physical exercise and lunch break), blood biochemical indicators (such as albumin, triglyceride, total cholesterol, high-density lipoprotein, low-density lipoprotein and fasting blood glucose), and medication usage (such as clozapine, olanzapine, risperidone, and chlorpromazine). Lastly, a neuropsychological test battery was used to assess their psychiatric and cognitive symptoms, for example, the Montreal Cognitive Assessment (MoCA) was used to assess their overall cognitive functioning. Their depressive symptoms were assessed by the geriatric depression scale (GDS). Activities of daily living (ADL) were used to assess their ability to lead a daily life, while the positive and negative syndrome scales (PANSS) were used to assess their psychiatric symptoms.

**Results:**

Smokers who develop schizophrenia at older ages had a higher body mass index than non-smokers. We also found that plasma albumin, triglycerides, low-density lipoprotein, and fasting blood glucose concentrations were significantly higher in smokers. In contrast, smokers with schizophrenia also had lower PANSS total scores, negative symptom scores, and general psychopathology scores. A forward stepwise binary logistics regression analysis demonstrated a significant association between negative symptom scores and smoking status (B = 0.112, *p* < 0.001, OR = 1.119, 95% confidence interval: 1.059–1.181). Correlation analysis was carried out and it was found that the amount of cigarette consumption per day had a negative correlation with plasma albumin level(r = − 0.290, *p* = 0.004). However, no such association was found in normal controls.

**Conclusions:**

Elderly Chinese men with schizophrenia have a higher percentage of smokers, and although smoking can reduce their plasma albumin levels, it does contribute to the prevention of negative symptoms.

## Introduction

Schizophrenia is a highly prevalent and severe mental illness that in many cases, results in a reduction in social and occupational status, which remains an etiological and therapeutic challenge [1]. Schizophrenia is characterized by distortions of thought and perception, without strict pathologic symptoms, and can be divided into different domains: positive, negative, and cognitive symptom domains [2]. Compared to the general population, people with schizophrenia have a shorter life expectancy, despite an increased risk of obesity, hypertension, hyperlipidemia, cardiovascular disease, and metabolic syndrome [3]. The onset of schizophrenia typically occurs in late adolescence or young adulthood. However, a growing body of evidence suggests that the disease results from gene-environment interactions that play a role in early life and neurodevelopment during adolescence [4].

Smoking is highly prevalent in mental illness and is especially prevalent in schizophrenia, with a prevalence of about 70–80% [5], and many of the morbidity and premature mortality in schizophrenia can be attributed to smoking-related illnesses. Furthermore, smokers also have an increased risk of schizophrenia [6]. In line with trends in the general population, male smokers with schizophrenia frequently smoke more heavily and more frequently than females [7]. Although a great deal of research has been conducted on the association between smoking and schizophrenia, the relationship remains controversial. For example, some researchers have suggested that smokers may be more likely to experience more intense positive symptoms, with a higher rate of recurrence and worse cognitive function, whereas others argue that smoking reduces adverse extrapyramidal reactions [8].

Because schizophrenia is a highly relapsing disease, many people with schizophrenia will transition to a chronic course [9]. Compared to first episode patients, patients with chronic schizophrenia often present with more unique clinical features, such as more positive symptomatology, less negative symptomatology, and lower levels of social support [10]. However, previous studies have tended to focus only on the association between cigarette smoking and first-episode schizophrenia, and relatively few studies have examined the association between cigarette smoking and chronic schizophrenia, and results have also been inconsistent. In Wei et al.’s study, for instance, they found that heavy smokers had fewer cognitive symptoms, suggesting that heavy smoking may be beneficial to cognitive function in patients with schizophrenia [11]. In another study, Wei et al. found that patients with chronic schizophrenia who smoke have more severe cognitive impairment than patients who do not smoke, especially in executive function and working memory [12]. In Kao et al.’s study, they found that smokers with chronic schizophrenia have higher rates of hospitalization, lifetime suicide attempts, antipsychotic side effects, psychopathology, impulsivity, depression, anxiety, and suicide risk than non-smokers with schizophrenia [13]. The differences between the studies mentioned above may be due to the different inclusion criteria and assessment instruments used.

Therefore, the aim of this study was to investigate the general demographics, biochemical indicators, clinical features, and cognitive function between smokers and non-smokers in Chinese Han patients with chronic schizophrenia. Given that only a small percentage of women with schizophrenia are smokers, we included only older men with chronic schizophrenia in the current study. We hypothesized that (1) smokers with schizophrenia would have less psychotic and cognitive symptoms than non-smokers and (2) smokers and non-smokers with schizophrenia may have different lipid levels.

## Methods

### Participants

Our study included 163 older men with chronic schizophrenia entered our study. All subjects were recruited from the geriatric ward of Shanghai Mental Health Center from 2021.1.1 to 2022.12.31. All subjects would be required to meet the following inclusion criteria: (1) meet DSM-IV diagnoses of schizophrenia confirmed by more than 2 treating psychiatrists based on the Structured Clinical Interview for DSM-IV (SCID); (2) 60 years of age or older; (3) Han Chinese; (4) progression of the disease is greater than 2 years of age; and (5) at least two episodes of illness. Exclusion criteria were: (1) patients with a first episode; (2) the condition was acute or unstable; (3) combined with organic brain disease or senile neurodegenerative disease; (4) substance dependence; and (5) patients or family members refused to participate in the study. All subjects meeting the enrollment criteria were required to complete a standardized questionnaire including gender, height, weight, years of education, age of onset, duration of disease, smoking status, disease status, lifestyle factors, use of medications, and so forth. For the purposes of the current study, we defined smokers as those who were daily smokers who had smoked more than 100 cigarettes in their lifetime and non-smokers as those who had never smoked or who had smoked less than 100 cigarettes in their entire lifetime [14]. The 163 subjects were then divided into two groups (smoker: *n* = 96 vs non-smoker, *n* = 67) according to whether or not they were a current smoker.

We also recruited 359 normal community elders at the same time to serve as normal control subjects, who had an average age of 71.27 ± 7.58 years and a mean educational attainment of 11.79 ± 3.60 years. Interviews were conducted by trained researchers with all healthy controls under the care of a research psychiatrist. No individual had a personal or family history of this condition, or a psychiatric evaluation for clinical mental health. They were classified as smokers (*n* = 190) and non-smokers (*n* = 169) according to whether they smoked or not.

All subjects signed informed consent prior to the study, and the study has been approved by the Ethics Committee of Shanghai Mental Health Center.

### Biochemical indexes

Peripheral blood samples were obtained between 7 and 9 am following an overnight fast. Biochemical Indexes were detected in the serum separator tube containing activated clot gel. Plasma albumin, triglyceride, total cholesterol, high-density lipoprotein, low-density lipoprotein, and fasting blood glucose were measured using an Olympus AU2700 automatic biochemical analyzer (Beckman Coulter, Inc., Carlsbad, CA, USA).

### Clinical and neuropsychological assessment

In the current study, experienced psychological raters were employed to perform a series of clinical assessments and neuropsychological tests on participants. The Montreal Cognitive Assessment (MoCA) [15] was used to assess their overall cognitive function. The Geriatric Depression Scale (GDS) was used to rule out depression [16]. The Activities of daily living scale (ADL) scale was used to assess the subject’s ability to function in everyday life [17]; and the Positive and Negative Syndrome Scale (PANSS) was used to assess the severity of psychotic symptoms in the participants [18]. Participants were asked to rate their psychotic symptoms.

### Statistical analysis

Continuous variables were expressed as means ± standard deviation and categorical variables were expressed as frequencies (%). The first step was to use a Kolmogorov–Smirnov test to test whether the data fit the normal distribution. An independent sample *t*-test was then used to compare normal data between smokers and non-smokers, whereas the Mann–Whitney *U* test was employed to compare non-normally distributed. The chi-square test was used to analyze the class variables between the two groups. The second part of the statistics used a generic linear (single variable) model to compare the biochemical indicators of blood and neuropsychological test results, while controlling for age of illness onset and BMI (due to statistical differences in age of onset and BMI in the smoking and non-smoking groups, both variables were controlled in subsequent statistics). We then conducted a binary stepwise logistics Regression Analysis (forward LR) to evaluate the relationship between smoking status (smoker vs non-smoker) and the other variables (illness onset age, BMI, albumin, triglycerides, high-density lipoprotein, low-density lipoprotein, fasting blood glucose, PANSS score, PANSS negative symptom scale, and PANSS general psychopathology). Lastly, correlation analysis was used to detect the correlation between the amount of smoking and the biochemical Indexes and each scale score. Two-sided tests were performed at the significance level of *p* < 0.05 and multiple comparison p values were corrected with a Bonferroni correction. SPSS version 22.0 (IBM, Armonk, NY, USA) was used for all statistical analyses.

## Results

### Subjects’ general demographic characteristics

Of the 163 older men with chronic schizophrenia, 58.9% of them were smokers (*n* = 96) and 41.1% were non-smokers (*n* = 67). We then compared the overall demographics of the 2 groups. Compared to non-smokers, smokers had a later age of onset of illness and a higher body mass index (*p* < 0.05). There was no significant difference between the two groups in terms of age, education, course of disease, hypertension, diabetes, hyperlipidemia, physical activity, lunch break, clozapine, olanzapine, risperidone, chlorpromazine, etc. The results are shown in Table 1. The mean age of smokers in the normal population was younger than that of non-smokers (67.82 ± 6.68 vs 72.68 ± 7.68, *p* < 0.01), and there were no statistical differences in years of education or BMI between the 2 groups.

**Table 1:** Comparison of general demographic data between smokers and non-smokers in elderly Chinese male patients with chronic schizophrenia

Items	Smoker (*n* = 96)	Non-smoker (*n* = 67)	F or X^2^	*p*
Age (years)	65.64 ± 5.42	67.28 ± 6.43	0.891	0.406
Education(years)	8.22 ± 2.96	8.04 ± 2.95	0.326	1.000
Duration of illness (years)	36.79 ± 11.41	39.95 ± 13.40	1.202	0.111
Age of illness onset (years)	28.81 ± 11.80	27.25 ± 11.94	1.401	0.039*
Duration of smoking (years)	29.76 ± 14.56	–	–	–
Number of cigarettes smoked	12.22 ± 7.37	–	–	–
Body Mass Index(kg/m2)	24.20 ± 3.87	22.76 ± 3.74	2.370	0.019*
Hypertension (%)	32 (33.3)	28 (41.8)	1.214	0.323
Diabetes (%)	20 (20.8)	8 (11.9)	2.194	0.205
Hyperlipidemia (%)	37 (38.5)	35 (52.2)	3.002	0.109
Lifestyle factors				
Physical exercise (%)	36 (37.5)	15 (22.4)	4.192	0.058
Lunch break (%)	82 (88.2)	38 (80.9)	1.367	0.307
Medication usage				
Clozapine (%)	17 (17.7)	9 (13.4)	0.538	0.520
Olanzapine (%)	20 (20.8)	17 (25.4)	0.463	0.570
Risperidone (%)	26 (27.1)	17 (25.4)	0.059	0.858
Chlorpromazine (%)	11 (18.3)	6 (18.2)	0	1.000

*p < 0.05

### Biochemical indexes and various scale scores

Compared to non-smokers, smokers had higher plasma concentrations of albumin, triglycerides, high-density lipoprotein, low-density lipoprotein, and fasting blood glucose, while PANSS total scores, PANSS negative symptom scores, and PANSS General psychopathology scores were lower. These results controlled for age disease onset and BMI. The results are shown in Table 2. There was no difference (*p* > 0.05) in MOCA, plasma albumin, cholesterol, high-density lipoprotein, low-density lipoprotein, fasting blood glucose, and triglycerides between smokers and non-smokers in the normal population (Table 3).

**Table 2:** Clinical, psychiatric, and cognitive features between smokers and non-smokers in elderly Chinese male patients with chronic schizophrenia

Items	Smoker (*n* = 96)	Non-smoker (*n* = 67)	F	*p*
Blood biochemistry				
Albumin (mmol/L)	41.01 ± 4.89	40.30 ± 3.54	2.872	0.038*
Triglyceride (mmol/L)	1.38 ± 0.78	1.19 ± 0.65	5.379	0.001*
Total cholesterol (mmol/L)	4.54 ± 0.92	4.34 ± 0.84	2.584	0.055
High-density lipoprotein (mmol/L)	1.22 ± 0.34	1.22 ± 0.43	8.103	< 0.001*
Low-density lipoprotein (mmol/L)	2.72 ± 0.78	2.54 ± 0.74	3.468	0.018*
Fasting blood glucose (mmol/L)	5.53 ± 1.34	4.95 ± 0.66	7.375	< 0.001*
Neuropsychological tests				
MoCA	15.79 ± 6.32	14.60 ± 7.07	2.259	0.085
GDS	9.98 ± 5.72	9.52 ± 6.62	0.182	0.908
ADL	23.57 ± 8.97	27.00 ± 10.72	1.993	0.118
PANSS				
Total scores	56.95 ± 16.04	68.46 ± 17.58	4.614	0.004*
Positive symptom scale	11.00 ± 4.59	11.72 ± 5.48	1.309	0.274
Negative symptom scale	15.97 ± 6.78	21.91 ± 8.16	6.547	< 0.001*
General psychopathology	29.84 ± 7.97	34.26 ± 8.99	2.906	0.037*

MMSE: Mini-mental State Examination; MoCA: Montreal Cognitive Assessment; GDS: Geriatric depression scale; ADL: Activities of daily living; PANSS: Positive And Negative Syndrome Scale; *p < 0.05

**Table 3:** Final result of binary stepwise logistics regression analysis (forward LR)

Item	B	S.E	Wald	df	*p*	OR	95% Confidence interval
Negative symptom scale	0.112	0.028	16.382	1	< 0.001*	1.119	1.059–1.181

*p < 0.05

### Links between smokers and biochemical indicators or various scale scores

**Fig. 1:**
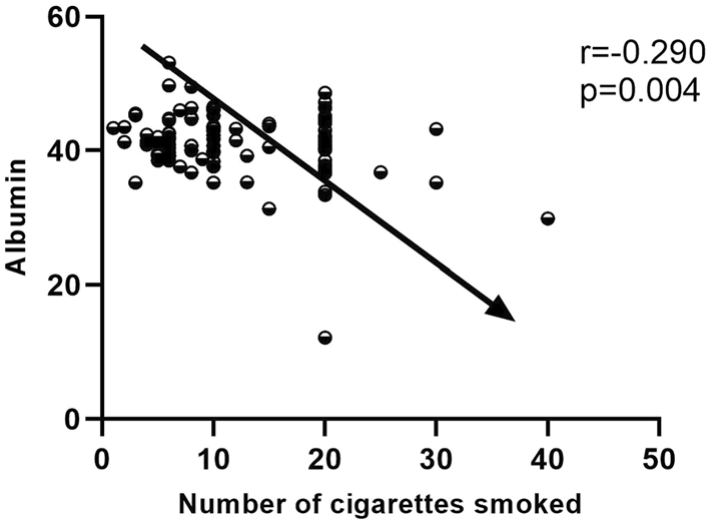
The association between number of cigarettes smoked per day and plasma albumin

A stepwise binary logistic regression analysis (forward LR) showed a significant correlation between smoking status and negative PANSS scale scores (B = 0.112, *p* < 0.001, OR = 1.119, 95%CI 1.059–1.181). By correlation analysis, we found a negative correlation between daily cigarette consumption and plasma albumin (r = − 0.290, *p* = 0.004). The results are shown in Fig. 1.

## Discussion

The aim of this study was to investigate the effects of smoking on blood biochemical indicators, cognitive function, and psychiatric symptoms in elderly Chinese patients with chronic schizophrenia. The results showed that (1) smokers account for a high proportion of elderly Chinese males with schizophrenia; (2) smoking has no effect on overall cognitive function in older men with chronic schizophrenia; (3) smoking was harmful to the health of elderly patients with schizophrenia but could effectively prevent negative symptoms; and (4) there was a negative correlation between daily cigarette consumption and plasma albumin in older men with chronic schizophrenia.

In this study, we found that smokers accounted for 58.9% of chronic schizophrenia in older men, compared to 54.1 percent in the normal control group. However, using the chi-square test, we found no difference in smoking rates between the 2 groups (X^2^ = 0.418, *p* = 0.546). In a study by Li and others, they found an 18.5% of tobacco use among people with schizophrenia [19]. In Zhang et al.’s study, they found that the prevalence of tobacco use among male patients with schizophrenia was 88% [20]. Another study showed that the smoking rate among Chinese males was 59% [21]. As a result, results vary widely from study to study and may be relevant to different topics. For example, some studies included patients with first episode schizophrenia, while others included older patients with chronic schizophrenia. In addition, some studies included only male patients, while others included female and male patients. Some studies have suggested that smoking and schizophrenia are more common than in the general population [22, 23], but we did not have uniform conclusions.

To assess the cognitive effects of smoking, we evaluate subjects’ MoCA scores. Instead, we found that smoking had no effect on overall cognitive function in older men with chronic schizophrenia. In Ringin et al.’s study, they found that smoking or not had no effect on cognitive function in schizophrenia patients [24]. In Ding et al.’s study, they found that smokers with schizophrenia were more likely to experience greater positive symptomatology and poorer cognitive function [8]. In a meta-analysis, it has been suggested that chronic smoking in schizophrenia is associated with cognitive impairments, particularly in learning, working memory, executive function, reasoning/problem resolution, and processing speed [25]. In addition, in a study by D’Souza et al., they found that smoking cessation could help reverse the cognitive damage caused by schizophrenia [26]. Therefore, it is necessary to further study the relationship between smoking and cognitive function in patients with schizophrenia.

While previous studies have suggested that smoking is harmful to the health of people with schizophrenia, it is worth noting that both univariate and multivariate analyses show that smoking helps reduce negative symptoms in older men with chronic schizophrenia. In the study of Smith RC et al., they found that acute tobacco use temporarily reduced negative symptoms in schizophrenia, but it was difficult to determine whether the effect was due to nicotine, other components of cigarettes or smoking behavior [27]. In Kotov et al.’s study, they found that tobacco use was not associated with psychiatric symptoms, but rather co-mutated with depression over time [28]. In addition, in Forchuk et al.’s study, they found that smoking may have a sedative effect and ameliorates negative symptoms [29]. Therefore, our conclusions are consistent. Potential mechanisms by which smoking ameliorates negative symptoms may involve its ability to promote social interaction, enhance attention and cognitive abilities, alleviate stress and depressive feelings, and produce feelings of pleasure [30].

We then sought to determine the relationship between daily smoking status and plasma biochemical indices. Through correlation analysis, we found that daily cigarette smoking was negatively correlated with plasma albumin concentration in the older male with chronic schizophrenia patient, but not in normal people. Albumin is a major source of total antioxidant capacity in human plasma, mainly due to its high concentration compared to other antioxidants in the blood, such as bilirubin, alpha-tocopherol, and beta-carotene [31]. Metal chelation may be involved in the antioxidant defense mechanism of plasma albumin. In addition to inhibiting lipid peroxidation by binding to copper ions, albumin can also act as oxygen and carbon centers to enhance the rate of scavenging free radicals [32]. Previous studies have shown that plasma albumin levels were significantly lower in patients with first episode schizophrenia, suggesting that patients with schizophrenia may have greater oxidative stress responses [33]. Similarly, it is often reported that patients with schizophrenia who smoke also have more oxidative stress reactions. Therefore, we hypothesize that smoking may increase the oxidative stress response by reducing the concentration of plasma albumin. Unfortunately, we did not link smoking status to albumin and negative symptoms, so smoking may not affect negative symptoms by affecting albumin levels.

We conclude by exploring the possible mechanisms for the effects of smoking on schizophrenia, and there can be many different perspectives based on findings from previous Mendelian randomization studies. On the one hand, there was strong evidence to suggest that smoking is a risk factor for both schizophrenia and depression [34]. In contrast, another Mendelian randomization study found that the associated single-nucleotide polymorphisms (SNPs) for all four tobacco use behaviors (such as smoking initiation, smoking cessation, age at smoking initiation, and quantity of smoking) were not significantly associated with schizophrenia status [35]. Thus, more longitudinal follow-up studies are required to further explore and test the relationship between cigarette smoking and schizophrenia.

We recognize that there are some limitations to our study. First, this was only a cross-sectional study and was unable to establish a causal association between cigarette smoking and negative symptoms of schizophrenia. Second, the relatively small sample size could also lead to some deviation from the research findings. Finally, we were unable to establish the relationship between smoking, plasma albumin, and negative symptoms, resulting in a failure to identify the biological mechanism by which smoking ameliorates negative symptoms.

## Conclusions

Older Chinese patients with schizophrenia are more likely to be smokers, and while smoking may be associated with lower plasma albumin levels, it may help to prevent negative symptoms. Thus, further research into the relationship between smoking and schizophrenia is warranted.
